# Classification of Infant Crying Sounds Using SE-ResNet-Transformer

**DOI:** 10.3390/s24206575

**Published:** 2024-10-12

**Authors:** Feng Li, Chenxi Cui, Yashi Hu

**Affiliations:** Department of Computer Science and Technology, Anhui University of Finance and Economics, Bengbu 233030, China

**Keywords:** SE-ResNet-Transformer, Mel-frequency cepstral coefficient (MFCC), classification of infant crying

## Abstract

Recently, emotion analysis has played an important role in the field of artificial intelligence, particularly in the study of speech emotion analysis, which can help understand one of the most direct ways of human emotional communication—speech. This study focuses on the emotion analysis of infant crying. Within cries lies a variety of information, including hunger, pain, and discomfort. This paper proposes an improved classification model using ResNet and transformer. It utilizes modified Mel-frequency cepstral coefficient Mel-frequency cepstral coefficient (MFCC) features obtained through feature engineering from infant cries and integrates SE attention mechanism modules into residual blocks to enhance the model’s ability to adjust channel weights. The proposed method achieved 93% accuracy rate in experiments, offering advantages of shorter training time and higher accuracy compared to other traditional models. It provides an efficient and stable solution for infant cry classification.

## 1. Introduction

Infant cry classification is a crucial and rapidly evolving branch within the broader field of speech classification. The act of crying serves as one of the most fundamental and instinctive forms of communication for infants, enabling them to convey a variety of essential needs and emotions. This primal vocal expression carries critical information, often indicating hunger, fatigue, discomfort, or even illness. However, unlike adult language, which is structured and widely understood, infant cries are often varied and nuanced, making it challenging for most caregivers, particularly young parents and those lacking experience, to accurately interpret their meanings. In a world that demands quick responses and effective solutions, it becomes imperative for caregivers to swiftly and accurately understand the information embedded in these cries. This understanding is vital not only for ensuring the physical health of the baby but also for promoting a happy and secure emotional environment essential for healthy development. Historically, many parents relied on anecdotal experiences and word-of-mouth wisdom passed down through generations to speculate about the meanings behind their infants’ cries. While this approach provided some insights, it often lacked precision and could not keep pace with the rapid changes and demands of modern parenting.

The evolution of infant cry classification can be broadly divided into two distinct stages: traditional machine learning-based methods and the more recent deep learning-based approaches. In the initial stage, researchers relied on traditional machine learning techniques to classify infant cries. These methods marked a significant advancement over earlier practices that depended solely on parental experience and subjective interpretation. For instance, in Mukhopadhyay’s [1] study, a trained group of individuals achieved a maximum classification accuracy of merely 33.09% in identifying different types of cries. In stark contrast, machine learning algorithms that utilized spectral and rhythmic features demonstrated a remarkable accuracy of 80.56% on the same dataset, highlighting the efficacy of algorithmic approaches in this domain.

The landscape of artificial intelligence has continually evolved [2], leading to the widespread adoption of various machine learning techniques in infant cry classification. Prominent methodologies such as hidden Markov models [3], support vector machines (SVMs) [4], and Gaussian mixture models [5] have shown considerable promise in accurately categorizing infant cries. Experimental results validate that these traditional machine learning techniques have significantly improved the classification tasks associated with infant cries, providing reliable frameworks for understanding this complex data.

However, the rapid advancements in big data and the diversification of data sources have introduced new challenges. The collection and classification of infant cry data have become increasingly intricate, resulting in enormously large and diverse datasets. Traditional machine learning methods, while effective to a degree, struggle to adapt to the evolving nature of these datasets. This inadequacy has paved the way for the emergence of deep learning-based infant cry classification research.

Deep learning approaches represent a significant leap forward in addressing the complexities and variations inherent in infant cry data. These methods harness the power of neural networks to process and learn from large volumes of data, allowing for improved classification accuracy and adaptability to different crying patterns. One key advantage of deep learning is its capacity to account for long-term dependencies within the cry data, which is crucial for understanding the subtleties of infant communication. In the deep learning model proposed in this paper, we integrate an improved Mel-frequency cepstral coefficient (MFCC) feature set, which enhances the ability to capture the acoustic properties of infant cries more effectively. The incorporation of advanced signal processing techniques enables a more nuanced understanding of the sounds produced by infants, allowing for better classification outcomes. Additionally, we introduce squeeze-and-excitation (SE) attention mechanism modules within the residual blocks of the ResNet architecture. This enhancement bolsters the model’s capability to adjust channel weights dynamically and select the most relevant features for classification, further refining the accuracy of the cry analysis.

The significance of this research extends beyond mere academic interest; it has profound implications for practical applications. By improving the accuracy of infant cry classification, caregivers can respond more effectively to the needs of their infants, ensuring timely interventions that can alleviate discomfort and address health issues. The potential for real-time analysis of crying patterns could revolutionize parenting practices, providing parents with tools that assist them in making informed decisions regarding their child’s well-being.

In a word, as we delve deeper into the realm of infant cry classification, it becomes clear that the integration of machine learning and deep learning technologies offers a promising pathway to enhancing our understanding of infant communication. By leveraging these advanced methodologies, we can create systems that not only reduce parental anxiety but also promote healthier developmental outcomes for infants. The future of this research is bright, with the potential to significantly impact the lives of families and contribute to the growing field of emotion analysis in artificial intelligence.

## 2. Related Works

Recently, research on the classification methods of infant cry signals has made significant progress. N. Nimbarte et al. [6] employed MFCC for feature extraction and K-nearest neighbors (KNNs) for classification, achieving high accuracy in cry-type recognition when tested on an open dataset. W. You et al. [7] employed long short-term memory (LSTM) networks for infant cry analysis. The model achieved a maximum accuracy of 92.39% in recognizing various types of infant cries and stabilized after 75 rounds. V. Bella et al. [8], besides combining MFCC and LSTM, introduced two data augmentation techniques, namely time stretching and pitch shifting, to improve model performance. B. Lv et al. [9] proposed an infant cry emotion recognition method based on a multiscale CNN-BLSTM (convolutional neural network–bidirectional long short-term memory network). N. Meephiw et al. [10] applied MFCC for feature extraction, studying and comparing different numbers of MFCC features to determine factors suitable for classification techniques. From the experimental results, MFCC:11 appeared to be well suited for infant cry classification. The results of Liang Y-C et al. [11] showed that CNN and LSTM both provided decent performance, around 95% in accuracy, precision, and recall, in differentiating healthy and sick infants. Micheletti et al. [12] also evaluated the deep learning model’s performance relative to LENA’s cry classifier, one of the most commonly used commercial software systems for quantifying child crying. Broadly, we found that both deep learning and LENA model outputs showed convergent validity with human annotations of infant crying. Yasin et al. [13] used machine learning and artificial intelligence to distinguish cry tones in real time through feature analysis. In the review by T. Özseven et al. [14], the methods used for the ICRC were summarized to guide future studies, and an overview of the developments was presented to the researchers. In addition, the findings of the studies examined were given comparatively. As a result of this study, it has been determined that the most used traditional methods in ICRC are MFCC as a feature set and neural network-based classifiers as classifiers. K. Zhang et al. [15] proposed a novel approach named BCRNet, which integrates transfer learning and feature fusion. The BCRNet model utilizes multidomain features as inputs and a transfer learning model to extract deep features. H. A. Patil et al. [16], through experiments on infant cry classification and speech emotion recognition tasks, extensively analyzed various attention-based methods. The results revealed that the transformer model surpassed the previous state-of-the-art level in infant cry classification, achieving a recall rate improvement of 10.9%. S. K. Singh et al. [17], by comparing the performance of CNN models and transformer models, categorized infant crying into five reasons: hunger, pain, hiccups, discomfort, and fatigue. Anders et al. [18] investigated convolutional neural networks (CNNs) for the classification of infant vocalization sequences. The target classes were ‘crying’, ‘fussing’, ‘babbling’, ‘laughing’, and ‘vegetative vocalizations’. The work by Sandhya, P., et al. [19] aims to identify the speaker in an emotional environment using spectral features and classify using any of the classification techniques to achieve a high speaker recognition rate. Feature combinations can also be used to improve accuracy. Matikolaie et al. [20] used a novel combination of short-term and long-term features from different timescales to develop an automatic newborn cry diagnostic system to differentiate the cry audio signals (CASs) of healthy infants from those with respiratory distress syndrome (RDS). P. Kulkarni et al. [21] presented a study on the classification of child cries based on various features extracted through speech and auditory processing. Certain spectral and descriptive features vary significantly in a child’s cry intended for a specific purpose. Ekinci et al. [22] interpret the information embedded in baby cry audio signals using sound processing methods and classify them using machine learning algorithms. To achieve this objective, they utilized a dataset consisting of baby cry audio signals divided into five distinct classes. Khosro Rezaee et al. [23] used automatic acoustic analysis and data mining in their study to determine the discriminative features of preterm and full-term infant cries. The approach proposed by Sutanto, Erwin, et al. [24] started with an analysis of the sound’s power from WAV files before exploring the 2D pattern, which includes features for the machine learning. From this work, around 85% accuracy could be achieved. The finest experimental results showed a mean accuracy of around 91% for most scenarios, and this exhibits the potential of the proposed extreme gradient boosting-powered grouped-support-vector network in neonate cry classification [25]. Matikolaie et al. [26] analyzed the CASs of newborns under two months old using machine learning approaches to develop an automatic diagnostic system for identifying septic infants from healthy ones. The ChatterBaby algorithm detected significant acoustic similarities between colic and painful cries, suggesting that they may share a neuronal pathway [27]. The audio and speech features (AS features) were exacted using a Mel–Bark-frequency cepstral coefficient from the spectrogram cry signal and fed into a DCNN. The output of the proposed system yielded a balanced accuracy of 92.31%. A highest accuracy level of 95.31%, highest specificity level of 94.58%, and highest sensitivity level of 93% were attained through the proposed technique [28]. A CNN architecture trained with recordings of babies from Australia was used for classifying the audio material of Romanian babies. This was in an attempt to see what happens should the participants belong to a different cultural landscape. The results of the CNN automatic classification were compared to those obtained by the Dunstan coaches. The conclusions have proved that Dunstan language is universal [29]. A method to extract cries is proposed by Cabon S et al. [30]. It is based on an initial segmentation between silence and sound events, followed by feature extraction on the resulting audio segments and a cry and non-cry classification.

Research on infant cry classification has advanced through various techniques. MFCC-based feature extraction, often paired with machine learning methods like KNN and SVM, has been widely used, as seen in the work of N. Nimbarte et al. and N. Meephiw et al. More recently, deep learning approaches, especially CNN and LSTM networks, have shown great promise. Studies by B. Lv, W. You, and V. Bella achieved high accuracy, with hybrid models like BCRNet and attention-based methods further enhancing performance. Data augmentation techniques, cross-cultural studies, and real-time classification have also contributed to significant improvements in the field.

This paper proposes an improvement upon the ResNet architecture by incorporating the squeeze-and-excitation (SE) attention mechanism module, resulting in the SE-ResNet-Transformer. It utilizes an enhanced version of Mel-frequency cepstral coefficients (MFCCs), comprising MFCCs, first-order differential coefficients of MFCCs, and second-order differential coefficients of MFCCs as features. Additionally, it establishes a database containing infant cry signals with three types of emotions by integrating the Donateacry-corpus, Chillanto database, and ESC-50 database.

## 3. Proposed Methodology

This section outlines the proposed infant cry emotion classification method in three parts. Dataset integration and preprocessing: The first step involves integrating the datasets and preprocessing the data. Feature engineering: The second step involves extracting MFCCs as input feature vectors. Construction of SE-ResNet-Transformer neural network: The third step involves building an SE multiscale ResNet-Transformer neural network, training it with the input feature vectors, and then using the trained model to predict test samples and calculate accuracy. The main innovations of this study are as follows: (1) Utilizing an enhanced version of Mel-frequency cepstral coefficients (MFCCs) composed of stacked MFCCs, first-order differential coefficients of MFCCs, and second-order differential coefficients of MFCCs as features; (2) constructing the SE-ResNet-Transformer model by incorporating the squeeze-and-excitation (SE) attention mechanism module and the encoder part of the transformer into the ResNet architecture; and (3) integrating multiple open-source infant cry datasets from different sources.

### 3.1. Preprocessing

This study creates a comprehensive database of infant cries representing three emotional states by combining the Donateacry-corpus, Chillanto database, ESC-50 database, and YouTube videos. Given the differences in labeling standards among the selected datasets, it is essential to standardize the labels and preprocess all files prior to feeding the data into the neural network. Utilizing infant cry datasets from various sources significantly improves the model’s generalization capability. According to the dataset label characteristics, the infant cries are classified into three categories: discomfort, hunger, and pain.

#### 3.1.1. Pre-Emphasis

Infant vocalizations are susceptible to interference from radiation emitted by the mouth and nose due to the characteristics of the infant sound system. While speech signals generally have higher energy in the low-frequency range, high-frequency components tend to decay quickly. As a result, the attenuation of these high-frequency components leads to a predominance of low-frequency sounds. To address this, pre-emphasis techniques are required. This approach not only enhances the high-frequency spectrum but also smooths out the entire frequency range, facilitating the analysis of sound parameters. In this study, infant cry signals are processed using a high-pass filter, which serves as the pre-emphasis filter. The processing of the signal *x*[*n*] is described by the following formula:

(1) y[n]=x[n]−αx[n−1]

The *α* value determines the frequency response of the pre-emphasis filter, with a larger α leading to greater high-frequency gain. Generally, selecting a value between 0.9 and 1 helps enhance high-frequency components while preserving signal balance and minimizing excessive distortion. Based on experimental comparisons, we have set *α* to 0.97 for this study.

#### 3.1.2. Framing

Fourier transformation is essential for analyzing the frequency distribution in infant cry audio. Since Fourier transformation requires the audio signal to be stable, framing and windowing operations are performed. Similar to speech signal analysis, “short-term analysis techniques” are the most appropriate for examining infant crying. Given the time-varying nature of cries, the analysis must rely on “short-term analysis”. In this study, a frame length of 20 ms is selected, as infant cry signals stabilize within the first 10 ms to 30 ms.

There are two common framing methods: continuous segmentation and overlap segmentation. This study opts for overlap segmentation, as it better preserves information continuity. The overlapping portion between consecutive frames, known as the frame shift, typically represents half of the frame length. 

#### 3.1.3. Windowing

During the framing process, the original signal may experience spectral leakage, leading to significant differences between the spectra of the original and framed signals. Windowing is an effective method to mitigate this leakage. When choosing a window function, two key factors should be considered: the shape of the window function and its length. There are various window function shapes, with the most commonly used being the Hamming window, Hanning window, and rectangular window.

The formula for the rectangular window is as follows:

(2) $$w(n)=\left\{\begin{array}{l} 1,0\leq n\leq N-1\\ 0,else \end{array}\right.$$

The formula for the Hanning window is as follows:

(3) $$w(n)=\left\{\begin{array}{l} 0.5\left[1-\cos\left[\frac{2\pi n}{\left(n-1\right)}\right]\right],0\leq n\leq N-1\\ 0,else \end{array}\right.$$

The formula for the Hamming window is as follows:

(4) $$w(n)=\left\{\begin{array}{l} 0.54-0.46\cos\left[\frac{2\pi n}{\left(n-1\right)}\right],0\leq n\leq N-1\\ 0,else \end{array}\right.$$

In the formulas above, *N* represents the length of the window.

While all three window functions exhibit low-pass characteristics in their frequency responses, the different shapes also influence the short-time characteristics after framing infant cries. Thus, the shape of the window function is crucial for time-domain analysis of baby crying signals. The rectangular window is favored for its spectral smoothness, making it widely used; however, it has the drawback of potentially losing high-frequency information, which is rich in the frequencies present in infant cries. Therefore, this study selects the Hamming window for the windowing operation.

### 3.2. Feature Engineering

MFCC is currently widely applied in the field of deep learning for audio processing. Because of the logarithmic way in which the human ear perceives frequency, it is susceptible to changes in the low-frequency range but less sensitive to changes in the high-frequency range. MFCC can effectively capture the nonlinear relationship between sound frequency and the human ear, making it capable of extracting various acoustic features from cry sound frequencies. The calculation formula for MFCC is as follows:

(5) $$Mel(f)=2595\mathrm{lg}(1+\frac{f}{700})$$

where lg is the logarithm with base 10.

#### 3.2.1. Power Spectrum

After framing and applying a windowing function, this paper utilizes the discrete Fourier transform (DFT) to convert the data, transforming the time-domain signal into its frequency-domain representation to obtain the power spectrum, denoted as *X*(*k*) in the following form:

(6) $$X(k)=\sum_{n=0}^{N-1}x(n)e^{\frac{-j2\pi nk}{N}},0\leq n,k\leq N-1$$

where the power spectrum *P*(*k*) is equal to the squared magnitude of the signal spectrum *X*(*k*), as shown in Equation (7). The power spectrum more accurately represents the energy characteristics of the cry signal, retaining some amplitude elements of the cry spectrum while discarding the phase characteristics of the cry spectrum, described as follows.

(7) $$P(k)=\frac{1}{N}\left|X{\left(k\right)}^{2}\right|$$

#### 3.2.2. Mel Filter Bank

The human ear can detect sound frequencies ranging from 20 Hz to 20,000 Hz, which is known as the audible frequency range. The sounds we perceive consist of a mixture of various frequencies. The Mel filter bank is graphically represented as a series of triangular filters. Typically, this set includes 20 to 40 ascending triangular filters, with each triangle’s starting point positioned at the midpoint of the previous triangle, reflecting a linear frequency distribution on the Mel scale—hence the name Mel filter bank. At each frequency, the product of *P*(*k*) and the filter *H_m_*(*k*) is calculated. The frequency response *H_m_*(*k*) of a triangular filter in the Mel filter bank is computed as follows:

(8) $$H_{m}(k)=\left\{\begin{array}{l} 0,k<f(m-1)\\ \frac{k-f(m-1)}{f(m)=f(m-1)},f(m-1)\leq k\leq f(m)\\ \frac{f(m+1)-k}{f(m+1)=f(m)},f(m)\leq k\leq f(m+1)\\ 0,k>f(m+1) \end{array}\right.$$

where the *m* represents the filter index, and *f* (*m* − 1), *f* (*m*), and *f* (*m* + 1) correspond to the start, middle, and end points of the filter, respectively. In the calculation, *m* ranges from 1 to 13. For a Mel triangular filter, *f* (*m*) represents the center frequency of the Mel triangular filter, *f* (*m* − 1) represents the starting point of the Mel triangular filter, and *f* (*m* + 1) represents the ending point of the Mel triangular filter.

By summing the total of *H_m_* (*k*), we obtain Equation (9), where the value of *M* is 13.

(9) $$\sum_{m=0}^{M-1}H_{m}(k)=1$$

#### 3.2.3. Logarithmic Spectrum

The logarithmic energy spectrum *S*(*m*) for each frame is obtained through logarithmic operations as follows:

(10) $$S(m)=\ln\left[\sum_{k=0}^{N-1}P(k)H_{m}(k)\right],0\leq m\leq M$$

where “ln” here represents the logarithm function with the natural logarithm base *e*.

#### 3.2.4. Discrete Cosine Transform

Discrete cosine transform (DCT) was performed on the logarithmic spectrum mentioned above to obtain the Mel-frequency cepstral coefficients (MFCCs) denoted as *C*(*n*), which represent the MFCC features. The corresponding equation is described as follows:

(11) $$C(n)=\sum_{m=0}^{N-1}S(m)\cos\pi n\frac{m-\frac{1}{2}}{M},n=1,2,\ldots ,L$$

MFCC captures the static characteristics of crying signals; however, the dynamic information of crying signals also contains rich features that can further improve accuracy. In order to capture the dynamic data of crying signals, this study calculate the first-order difference coefficients *D*(*n*) and the second-order difference coefficients *D*_2_(*n*) based on MFCC. The formulas are as follows:

(12) $$D(n)=\frac{1}{\sqrt{\sum_{i=-k}^{i=k}i^{2}}}\sum_{i=-k}^{i=k}i\cdot C(n+i)$$

(13) $$D_{2}(n)=\frac{1}{\sqrt{2\sum_{i=-k}^{i=k}i^{2}}}\sum_{i=-k}^{i=k}i\cdot D(n+i)$$

**Figure 1:**
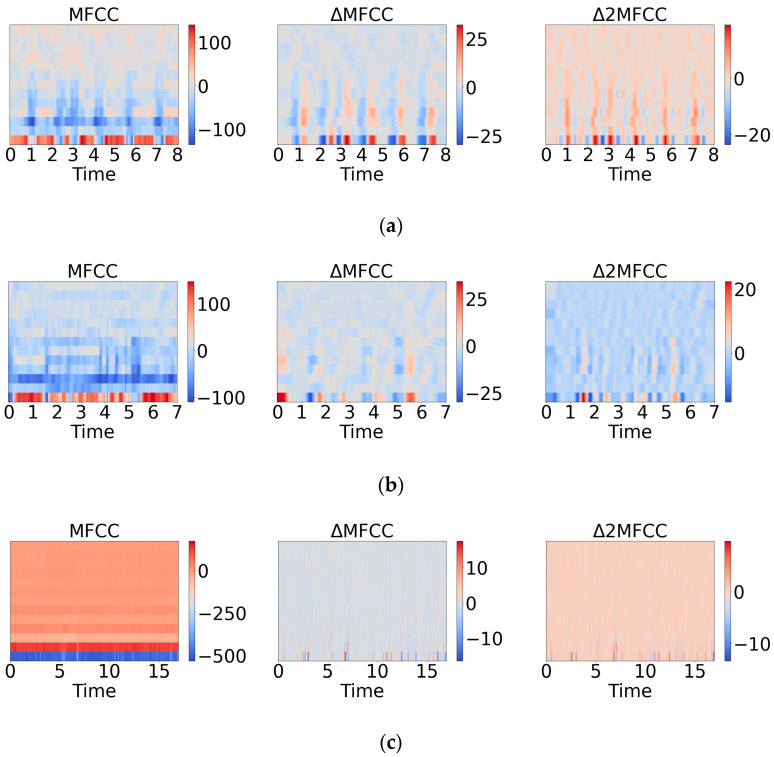
(**a**) Feature map of pain. (**b**) Feature map of hunger. (**c**) Feature map of uncomfortable.

The *k* is set to 2, and *C* (*n* + *i*) represents the Mel-frequency cepstral coefficients of a frame. Figure 1 displays their two-dimensional visualizations., where ΔMFCC is the result of Equation (12), and Δ2MFCC is the result of Equation (13). They are all of size (128, 40), which we use to construct a feature of size (128, 120) as input for the neural network. The extracted features are depicted in the following figure.

### 3.3. Model Building

#### 3.3.1. Residual Neural Network

**Figure 2:**
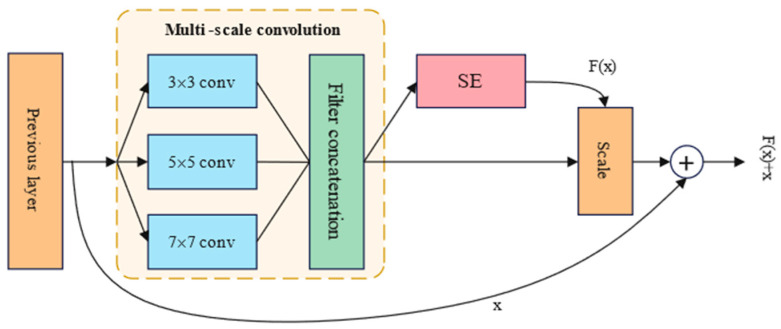
Structure of residual block.

The operational concept of classical convolutional neural networks (CNNs) involves converting raw data into a two-dimensional matrix structure, which surpasses conventional artificial neural networks in image manipulation and feature identification. However, as the network depth increases, the model’s accuracy initially improves continuously, reaching a maximum saturation point. With further increase in network depth, instead of continuing to increase, the model’s accuracy may significantly decrease, resulting in higher errors in both the training and testing phases compared to shallower models. This phenomenon arises from issues like gradient explosion and vanishing gradients, stemming from the escalating depth of preceding network architectures. Residual neural networks (ResNets) were originally proposed by He et al. Based on traditional convolutions, ResNet introduces the concept of residual blocks. As the deep neural network advances, the information gathered at each layer progressively decreases. ResNet effectively addresses this issue through the identity mapping of residual blocks, as illustrated in Figure 2. The subsequent layer not only encompasses the data from the present layer but also integrates fresh information acquired through nonlinear transformation. The principle of residual blocks is represented by the equation H(x) = F(x) + x, where F(x) = H(x) − x. Here, y = x represents the observed value, H(x) represents the predicted value, and, thus, H(x) − x represents the residual, which is F(x). This processing technique enables information to accumulate layer by layer, alleviating worries about data loss. Residual blocks, intuitively, maintain data integrity by directly transferring input information to the output, ensuring its preservation.

#### 3.3.2. SE Attention Mechanism

**Figure 3:**
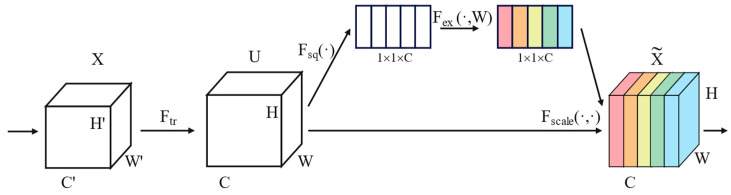
Structure of SENet.

Squeeze-and-excitation network (SENet) is a novel network architecture proposed by Hu et al. The core idea of SENet is to learn feature weights based on the loss during network training, such that effective feature channels have large weights while ineffective or less effective feature channels have small weights. This enables the model to achieve better results. The structure of SENet is illustrated in Figure 3.

SENet is not a complete neural network but rather a substructure that can be embedded into other classification models such as ResNet. This article constructs by embedding the SE attention mechanism module into some residual blocks of ResNet. SENet mainly consists of the following three operational steps:

(1) Squeeze

This operation compresses each *H* × *W* dimensional feature map into a single real number *z_c_* with global spatial information, as shown in Equation (14).

(14) $$z_{c}=F_{sq}(t_{c})=\frac{1}{H\times W}\sum_{i=1}^{W}\sum_{j=1}^{H}t_{c}(i,j)$$

Here, *t_c_* represents the channel descriptor of the input feature map, while *i* and *j* represent the indices of the channel and spatial dimensions, respectively.

(2) Excitation

In this step, the obtained *z_c_* is fed into a fully connected layer, and the resulting output is then passed through a ReLU activation function. Subsequently, it goes through another fully connected layer and finally passes through a sigmoid activation function, as shown in Equation (15).

(15) $$s=F_{ex}(z,W)=\sigma (g(z,W))=\sigma (W_{2}\delta (W_{1}z))$$

Here, *δ* represents the ReLU activation function, *σ* represents the Sigmoid activation function, and *W*_1_ and *W*_2_ represent the fully connected layers, where the dimension of *W*_1_ is C/r × C and the dimension of *W*_2_ is C × C/r. Since the dimension of *z* is 1 × 1 × C, the dimension of *W*_1_*z* is 1 × 1 × C/r. Then, it passes through a ReLU activation function, keeping the output dimension unchanged. Afterward, it is multiplied by *W*_2_, resulting in an output dimension of 1 × 1 × C. Finally, it passes through the sigmoid function to obtain *s*, which represents the weights of each channel feature map. Here, r is a scaling parameter, which is set to 16, 32, 64, and 128 in different SE convolutional layers of this model. The purpose of this parameter is to reduce the number of channels and hence computational complexity.

(3) Reweight

This step multiplies the obtained channel weights *s_c_* with the feature map matrix uc channel-wise to obtain the weighted feature map $\tilde{t_{c}}$. It is represented as Equation (16).

(16) $${\tilde{t}}_{c}=F_{scale}(u_{c},s_{c})=s_{c}u_{c}$$

where *F_scale_* represents the element-wise multiplication of the channel weights *s_c_* and the feature map matrix *u_c_* along the channels.

#### 3.3.3. Transformer

**Figure 4:**
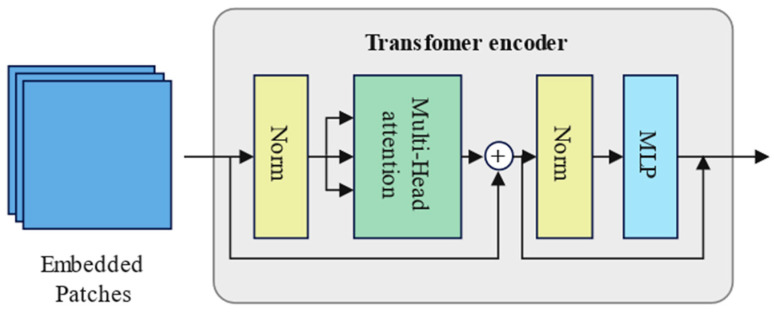
Structure of the encoder part of the transformer.

To better learn the long-term features in infant crying, this study introduces the transformer to facilitate capturing long-range dependencies. However, in classification tasks, typically only the encoder part of the transformer is used, as shown in Figure 4. In addition, this study employs a multilayer multihead attention mechanism with different parameters, enabling our model to learn the features of infant crying more effectively.

#### 3.3.4. SE-ResNet

**Figure 5:**
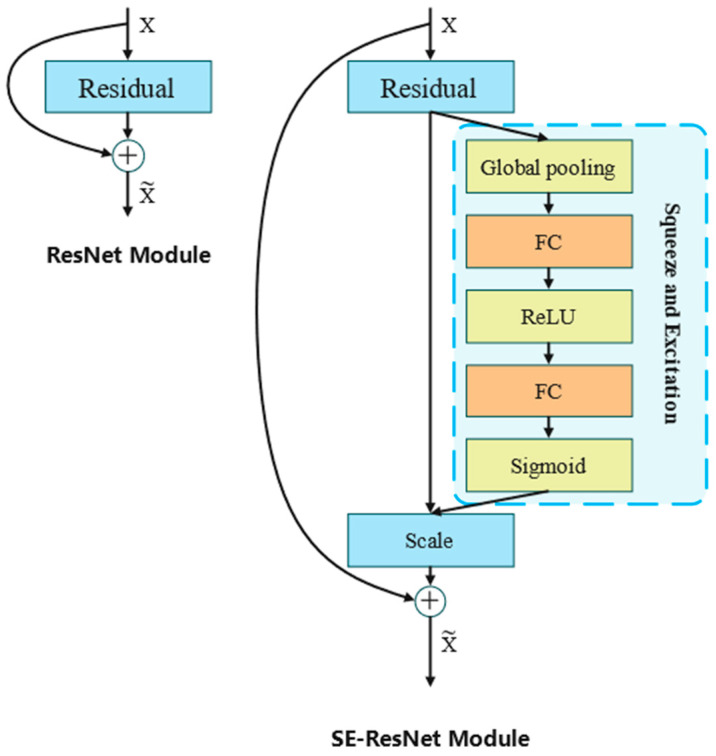
Structure of original residual module and SE-ResNet module.

This article constructs the SE-ResNet neural network by incorporating residual blocks containing SE attention mechanism modules into the ResNet architecture. These residual blocks with SE attention mechanism modules are referred to as SE residual blocks. By adding SE attention mechanism modules to the residual blocks of ResNet, the model is encouraged to learn more important information from the feature maps, thereby improving the model’s ability to adjust channel weights and adaptively select channels. It is important to note that in this article, the SE attention mechanism recalibrates the features of the residual branches before the addition operation, rather than recalibrating the features of the main branch after the addition operation. This is because there is a scale operation in the main branch, which ranges from 0 to 1. During backpropagation optimization in deeper layers of the network, gradient vanishing may occur near the input layer, making it difficult to optimize the model. The structure of the SE residual block is illustrated in Figure 5.

#### 3.3.5. SE-ResNet-Transformer

**Figure 6:**
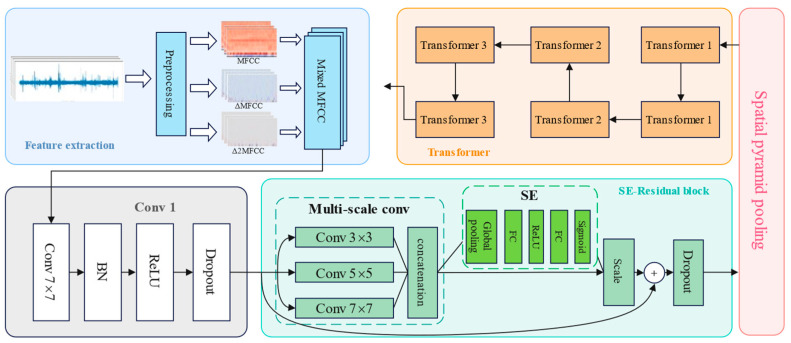
The workflow of the proposed method in this study.

This study builds SE-ResNet by integrating the SE attention mechanism into ResNet, and the feature information extracted by SE-ResNet is fed into the encoder part of the transformer. Finally, infant crying is classified, and the structure of SE-ResNet-Transformer is shown in Figure 6.

The network architecture of the SE-ResNet-Transformer neural network constructed in this article, which is the ResNet neural network with SE residual blocks and a transformer added, is shown in Table 1.

### 3.4. Combine Datasets

In order to enhance the model’s generalization capability, this article established a baby cry dataset containing three types of emotions by integrating the Donateacry-corpus, Chillanto, and ESC-50 databases in Table 2.

(1) Donateacry-corpus:

This dataset is sourced from a GitHub philanthropic project. The repository of this project contains raw, unmodified, and unchecked audio samples uploaded by users through the Donate-a-cry mobile application (available for Android and iOS). Additionally, the authors have labeled some of the data. This dataset comprises a total of 457 baby cry samples, as detailed in the table below.

(2) Chillanto:

The Baby Chillanto database is compiled by the CONACYT National Institute of Astrophysics, Optics, and Electronics in Mexico. It comprises five categories of cry signals: deafness, suffocation, normal, hunger, and pain. Each cry signal is standardized to one second in duration, resulting in a total of 2268 cries.

(3) ESC-50:

The ESC-50 dataset consists of approximately two thousand audio recordings, each containing fixed-length 5-second segments. There are a total of 50 types, with 40 samples per type, further divided into five different main categories.

Since the selected datasets have different classification labels and the data volumes under each label are not balanced, directly aggregating the datasets to construct a new dataset may result in low accuracy due to issues such as too many multiclass categories and data imbalance. Hence, we conducted a statistical analysis of the number of data samples under different labels in each dataset. We then integrated a series of common and small-sized baby discomfort behaviors, such as tiredness, needing to be held, hiccupping, loneliness, and fear, into a new label: “uncomfortable”. Additionally, we grouped a large number of hunger-related synonyms such as hungry, need to eat, and want something to eat into a unified new label: “hungry”. Furthermore, we noticed that there is a considerable amount of data related to illnesses in each dataset, such as abdominal pain, headaches, and oral pain. We believe that cries reflecting such conditions require more immediate attention from parents or other caregivers to ensure the baby’s health. Therefore, this article also established a relatively small-sized “pain” label.

The integrated dataset in this article contains approximately five thousand baby cry samples, with 20% of the data allocated to the test set and the remaining 80% to the training set. A random seed was used to ensure the consistency of the partitioning results. The establishment of this mixed dataset effectively addresses the current lack of large-capacity publicly available baby cry datasets on the internet.

## 4. Experimental Evaluation

### 4.1. Experimental Environment

The experimental environment for this study is shown in the Table 3.

### 4.2. Experimental Design

To investigate whether the proposed mixed MFCC better reflects the effective feature information in infant cry sounds compared to the original MFCC and its differential coefficients, we conducted experiments using the mixed MFCC, MFCC, MFCC first-order differential coefficients, and MFCC second-order differential coefficients as features. To explore the impact of integrating the SE attention mechanism module into ResNet, under consistent hyperparameters, we compared the model proposed in this paper with ResNet18, ResNet34, SE-ResNet34, ResNet50, and SE-ResNet50. The epochs were uniformly set to 50 and the batch size to 64, an Adam optimizer was used, and the learning rate was adjusted to 0.0001.

To verify that the residual mechanism and SE mechanism introduced in this study contribute to better classification performance, we conducted ablation experiments on the proposed method. Under the premise of using the same dataset, the same feature extraction, and the same hyperparameters, we compared the precision, recall, and F1 score of different models. To verify that the multiscale convolution used in this study can effectively improve the classification of infant cries compared to ordinary convolution, we conducted comparative experiments.

To explore the effects of transformer encoder models with different parameters on infant cry classification, we conducted experiments on Transformer 1, Transformer 2, and Transformer 3 while keeping the number of encoder layers constant. To validate the effectiveness of the neural network model proposed in this study for infant cry classification, we compared the classification results of different models under the premise of using the same features. To explore the impact of different reductions in the SE attention mechanism used in this study on the model’s classification performance, we conducted comparative experiments. Since the number of channels in the SE-Residual module is 64, we set the reductions to 16, 32, and 64 for comparison.

### 4.3. Evaluation Criteria

In this study, accuracy, precision, recall, F1 score, and training time are used as comprehensive metrics to assess the performance of the models. Since the dataset constructed in this study has relatively balanced labels, the overall performance metrics are calculated as macro-averages, which do not distinguish between labels.

(17) $$Accuracy=\frac{TP+TN}{TP+FP+TN+FN}$$

(18) $$Precision=\frac{TP}{TP+FP}$$

(19) $$Recall=\frac{TP}{TP+FN}$$

(20) $$F1=2\cdot \frac{Precision\cdot Recall}{Precision+Recall}$$

### 4.4. Experimental Result

**Figure 7:**
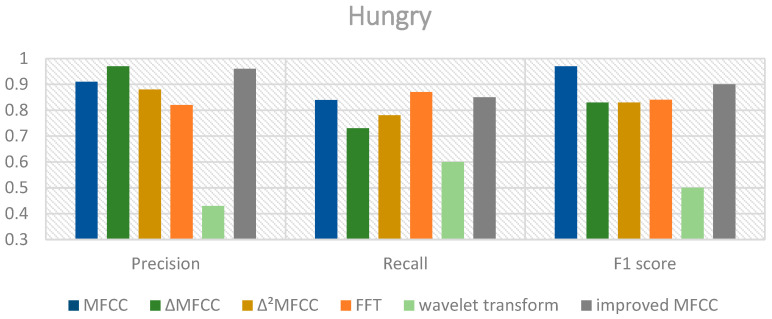
Performance of different features of hungry cries.

**Figure 8:**
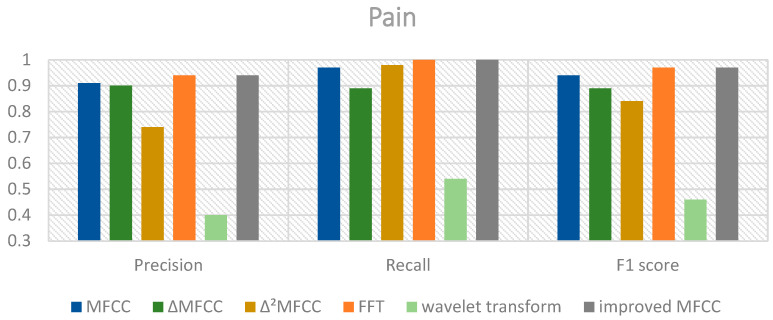
Performance of different features of pain cries.

**Figure 9:**
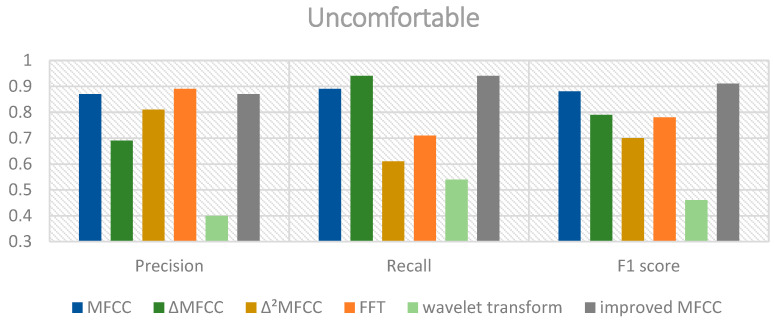
Performance of different features of uncomfortable cries.

The classification results using the mixed MFCC, MFCC, MFCC first-order differential coefficients, and MFCC second-order differential coefficients as features are shown in Figure 7, Figure 8 and Figure 9.

From the above charts, it can be observed that the performance of the mixed MFCC is slightly weaker in terms of precision and F1 score for the hungry label compared to other features. This indicates that the mixed MFCC as a feature can indeed effectively improve the classification performance of the model. Furthermore, the SE-ResNet-Transformer using the mixed MFCC demonstrates excellent classification performance for the pain label, which would be beneficial for caregivers to quickly and accurately detect the infant’s pain condition and respond promptly. The classification accuracies of the proposed model and ResNet18, ResNet34, SE-ResNet34, ResNet50, and SE-ResNet50 are shown in Table 4.

By comparing the data of different ResNets, it can be observed that integrating the SE attention mechanism into ResNet indeed significantly enhances the model’s classification performance for infant cry sounds. ResNet34 already demonstrates decent classification performance, but SE-ResNet34, constructed with the inclusion of the SE attention mechanism module, exhibits superior classification capabilities compared to other models. The experiment finally achieved better classification results by adjusting the complexity of the network structure, resulting in an optimized SE-ResNet-Transformer.

The effectiveness of the SE attention mechanism and residual blocks introduced in this study for classifying infant cries was significantly improved, as verified through ablation experiments. The results of the ablation experiments are shown in Table 5.

From the table above, it can be seen that the method proposed in this study shows significant improvements in all metrics compared to the original model without the SE mechanism and residual blocks. Both the SE mechanism and residual blocks contribute to enhancing the model’s performance to a certain extent. The experimental results clearly show that the multiscale convolution used in this study can effectively improve classification accuracy in Table 6.

By comparing encoder layers with different parameters while keeping the number of encoder layers constant, we found that the multilayer encoder with varying parameters used in this study offers a slight advantage in classification accuracy in Table 7. Additionally, we observed that even with fewer parameters, the accuracy remained relatively high, along with reduced computational complexity, making it more suitable for deployment. This provides direction for our future research.

**Figure 10:**
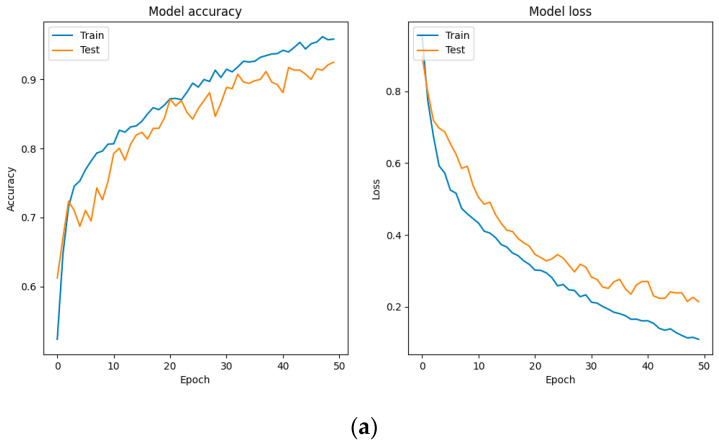

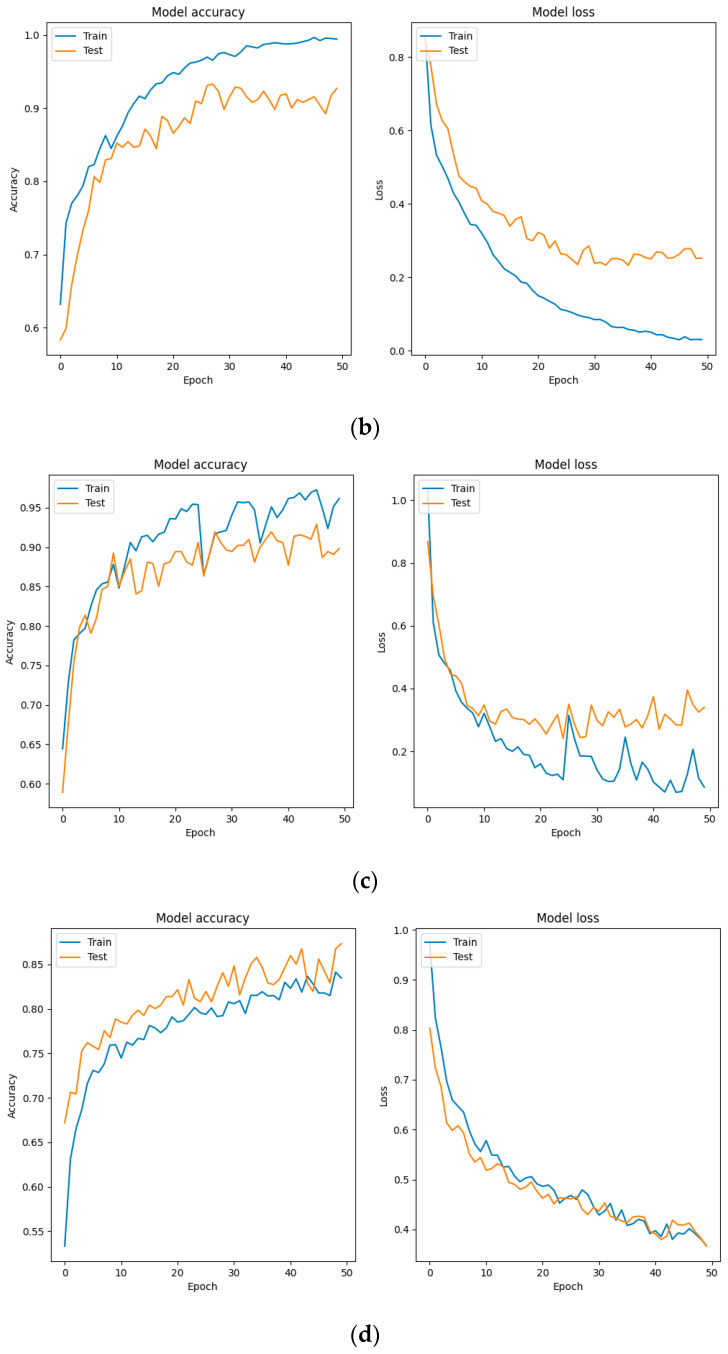

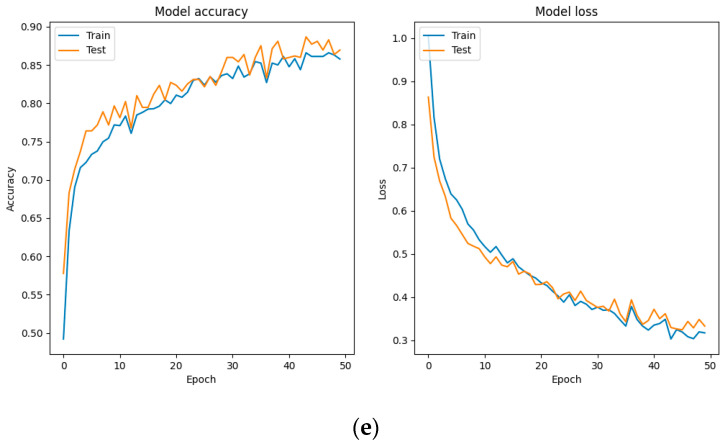
(**a**) Proposed method; (**b**) CNN-Transformer; (**c**) CNN; (**d**) GRU; (**e**) LSTM.

The following are the results of different models when using mixed MFCCs as features in Figure 10.

By comparing the images above, we can conclude that the method proposed in this study effectively addresses the overfitting issue of the previously proposed CNN-Transformer model while maintaining accuracy. Moreover, compared to traditional models such as CNN, GRU, and LSTM, the proposed method demonstrates significant advantages in both accuracy and convergence performance. The following are the results of this study exploring the impact of different reductions on the classification performance of infant cries.

**Figure 11:**
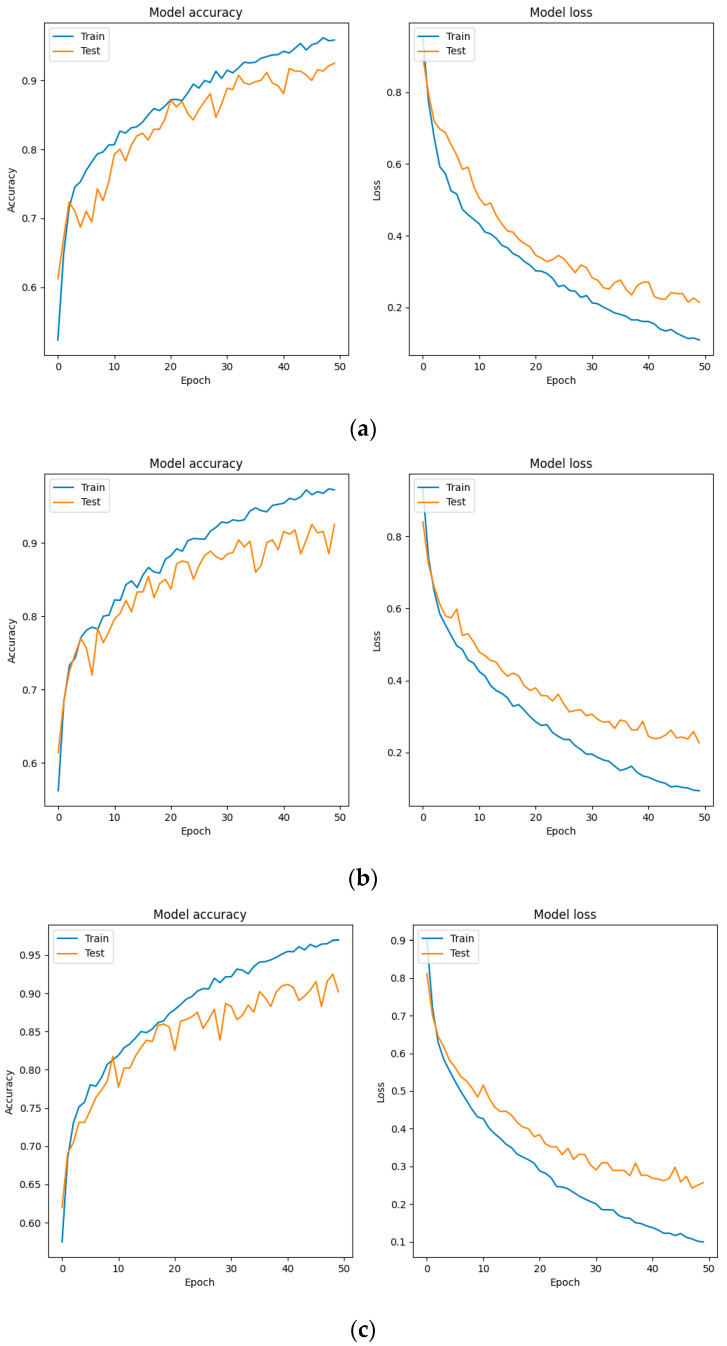
(**a**) The reduction is 16; (**b**) the reduction is 32; (**c**) the reduction is 64.

As shown in Figure 11, a larger reduction leads to more severe overfitting issues. Therefore, this study uses a smaller reduction (16) to enhance the model’s ability to capture nonlinear features in MFCC, resulting in better classification performance.

In addition, the results of this article are also compared with those of recent studies using the same datasets as this article, such as ESC-50, Baby Chillanto, etc. Since the dataset used in this article is a self-built mixed dataset, and most recent studies have not open-sourced their code, this article can only compare the final classification accuracy with them, as shown in Table 8.

## 5. Conclusions

This paper addresses the scarcity of publicly available infant cry sound datasets by integrating data from multiple platforms, thereby providing a stable foundation for neural network training. Additionally, the use of the mixed MFCC as a feature is demonstrated through experiments to significantly enhance the model’s performance. The proposed method of integrating the SE attention mechanism and transformer into ResNet enables the model to learn more important information from the feature maps, thereby enhancing the model’s ability to adjust channel weights and adaptively select channels. The proposed model demonstrates a 93% accuracy rate on the developed dataset, and effectively addresses the overfitting issue of the previously proposed CNN-Transformer model while maintaining accuracy, highlighting the efficacy of the suggested method for classifying infant cry sounds.

## Figures and Tables

**Table 1:** The network structure of SE-ResNet-Transformer.

Layer Name	Layer
Conv	7 × 7, 64, stride 2
SE-Multi Scale Residual Block	$\mathrm{Concatenate}\;\left[\begin{array}{c} 3\times 3,\;64\\ 5\times 5,\;64\\ 7\times 7,\;64 \end{array}\right]$ fc,[16,64]
Spatial Pyramid Pooling	[1, 2, 4]
Transformer 1	Head size = 16, Head number = 4, Feed-forward dim = 32, Layers = 2
Transformer 2	Head size = 32, Head number = 8, Feed-forward dim = 64, Layers = 2
Transformer 3	Head size = 64, Head number = 816, Feed-forward dim = 128, Layers = 2
	Average pool, 1000-d fc, softmax

**Table 2:** Mixed dataset.

Type	Number
Hungry	2080
Uncomfortable	1760
Pain	1364

**Table 3:** Experimental environment of this study.

Configuration Name	Configuration Information
Operating system	Win11
Development language	Python3.9
Framework	Tensorflow2.15.0
CPU	12th Gen Intel(R) Core (TM) i7-12700H 2.70 GHz
GPU	GeForce RTX 3060(6G)
RAM	16G

**Table 4:** Accuracy of different modules.

Neural Network	Accuracy
SE-ResNet-Transformer	93%
SE-ResNet34	92%
ResNet18	87%
ResNet34	88%
ResNet50	84%
SE-ResNet50	87%

**Table 5:** Ablation experiments of the newly introduced mechanism.

Neural Network	Precision	Recall	F1 Score	Accuracy
SE-ResNet-Transformer	0.92	0.93	0.92	0.93
ResNet-Transformer	0.91	0.91	0.91	0.91
SE-Conv-Transformer	0.91	0.91	0.91	0.91
Conv-Transformer	0.89	0.89	0.89	0.89

**Table 6:** Comparison experiments of different convolutional layers.

Neural Network	Precision	Recall	F1 Score	Accuracy
SE-ResNet-Transformer (multiscale)	0.92	0.93	0.92	0.93
SE-ResNet-Transformer (ordinary)	0.88	0.88	0.89	0.88

**Table 7:** Comparison experiments of encoder layers with different parameters.

Neural Network	Precision	Recall	F1 Score	Accuracy
Proposed method	0.92	0.93	0.92	0.93
Transformer 1	0.92	0.92	0.92	0.92
Transformer 2	0.92	0.91	0.91	0.91
Transformer 3	0.92	0.91	0.92	0.92

**Table 8:** Comparison with the latest research.

References	Features	Classifiers	Accuracy
Muhammad [31]	MFCC	CNN	86%
Qiao [32]	Spectrogram, Mel-scaled spectrogram, MFCC	AlgNet	87%
Zhang [33]	LFCC, MFCC	VGG16, LSTM	90%
Proposed	Mixed MFCC	SE-ResNet-Transformer	93%

## Data Availability

The datasets generated and analyzed during the current study are available from the corresponding author on reasonable request.
